# A passive mechanism for decoupling energy storage and return in ankle–foot prostheses: A case study in recycling collision energy

**DOI:** 10.1017/wtc.2021.7

**Published:** 2021-07-28

**Authors:** Hashim A. Quraishi, Max K. Shepherd, Leo McManus, Jaap Harlaar, Dick H. Plettenburg, Elliott J. Rouse

**Affiliations:** 1 BioMechanical Engineering Department, Delft University of Technology, Delft, The Netherlands; 2 Department of Mechanical Engineering and Robotics Institute, University of Michigan, Michigan, USA; 3 Neurobionics Lab, University of Michigan, Michigan, USA; 4 Department of Biomedical Engineering, Northwestern University, Illinois, USA

**Keywords:** amputation, ankle, biomechanics, energy recycling, gait, foot, prosthesis

## Abstract

Individuals with lower limb amputation experience reduced ankle push-off work in the absence of functional muscles spanning the joint, leading to decreased walking performance. Conventional energy storage and return (ESR) prostheses partially compensate by storing mechanical energy during midstance and returning this energy during the terminal stance phase of gait. These prostheses can provide approximately 30% of the push-off work performed by a healthy ankle–foot during walking. Novel prostheses that return more normative levels of mechanical energy may improve walking performance. In this work, we designed a Decoupled ESR (DESR) prosthesis which stores energy usually dissipated at heel-strike and loading response, and returns this energy during terminal stance, thus increasing the mechanical push-off work done by the prosthesis. This decoupling is achieved by switching between two different cam profiles that produce distinct, nonlinear torque–angle mechanics. The cams automatically interchange at key points in the gait cycle via a custom magnetic switching system. Benchtop characterization demonstrated the successful decoupling of energy storage and return. The DESR mechanism was able to capture energy at heel-strike and loading response, and return it later in the gait cycle, but this recycling was not sufficient to overcome mechanical losses. In addition to its potential for recycling energy, the DESR mechanism also enables unique mechanical customizability, such as dorsiflexion during swing phase for toe clearance, or increasing the rate of energy release at push-off.

## Introduction

The ankle joint plays a critical role during gait, absorbing energy during collision with the ground, contributing to overall stability, and providing the majority of net positive work for the forward propulsion of the body (Winter, 1991; Farris and Sawicki, 2011; Zelik et al., 2015). Individuals with transtibial amputation suffer from decreased late stance push-off work from the ankle joint, for which other joints must compensate. These compensations lead to an increased cost of transportation and can lead to greater stresses in the knee, hip, and contralateral ankle joint, causing long-term health issues and comorbidities due to asymmetrical joint loadings among other causes (Sanderson and Martin, 1997; Nolan and Lees, 2000; Silverman et al., 2008).

Energy storage and return (ESR) feet are passive prostheses capable of storing elastic energy during midstance and returning it during late stance to help transition the center of mass over the leading limb (Casillas et al., 1995; Hafner, 2006; Versluys et al., 2009). While ESR feet can restore approximately 30% of the push-off work done during late stance (Bovi et al., 2011), this is substantially less than the push-off work provided by healthy ankle musculature during each step. Further limitations of their ability to passively emulate the healthy ankle–foot complex include a softening behavior as the center of pressure shifts anteriorly during midstance, which opposes the stiffening behavior seen in the intact ankle, and the inability to actively dorsiflex the foot to achieve a desired toe clearance during swing phase (Winter, 1991; Rouse et al., 2014).

Powered prostheses can increase push-off work and regulate the ankle mechanics in a more controlled manner than ESR feet (Au et al., 2009; Wang et al., 2015; Gabert et al., 2020), but have several drawbacks that have reduced their transition into clinical practice, including higher mass, complexity, build height, and cost. Alternatively, “quasi-passive” prostheses can be used to enhance the capabilities of passive designs, while mitigating drawbacks related to powered devices. The governing principle of quasi-passive prostheses is to use small actuators to adjust the passive mechanics (such as stiffness, set-point, or damping) during the swing phase of gait (Lee et al., 2017; Shepherd and Rouse, 2017b; Glanzer and Adamczyk, 2018). Energy generated by these motors is not injected in the gait cycle, thereby allowing the use of smaller and quieter motors. By modulating passive mechanics, the prosthesis can adapt to multiple ambulatory tasks and walking speeds.

In previous research, passive and quasi-passive prosthetic technologies have been developed that implement cams, clutches, and ratchets to address several of the abovementioned limitations of ESR feet. Cam profiles have been used to replicate the nonlinear ankle mechanics during early and midstance (Realmuto et al., 2015; Lenzi et al., 2017; Shepherd and Rouse, 2017a; Shepherd and Rouse, 2017b). Clutches have been used to adjust the range of ankle mechanics (Lee et al., 2017) to enhance the push-off near late stance (Collins and Kuo, 2010; Rice et al., 2016), and to passively adapt to surface slopes during walking by effectively varying the equilibrium angle of the elastic mechanism (Nickel, 2014). Furthermore, researchers have increased the range of motion during push-off by utilizing a ratcheting mechanism in combination with a planetary gear transmission (Brackx et al., 2013). These works describe novel techniques and mechanisms that enable the exploration of ankle mechanics beyond the capabilities of nonlinear springs.

In this work, we extend our previous research on cam-based transmissions in prostheses, and introduce a lightweight mechanism that enables passive decoupling of the ankle prosthesis torque–angle mechanics. This mechanism is designed for integration in the variable stiffness prosthetic ankle (VSPA) foot (Shepherd and Rouse, 2017b), but could, in theory, be implemented in other prosthetic feet designs as well. The mechanism comprises two distinct cam profiles that interface with a leaf spring that is deflected upon rotation of the ankle joint. The desired ankle mechanics can be encoded in the shape of each cam profile, and by interchanging the cam profiles at specific points during the stance phase of gait (i.e., points in the torque–angle relationship), the ankle prosthesis can produce multiple energy storage and return profiles. Where conventional passive prosthetic feet can produce nonlinear ankle mechanics, this decoupling mechanism provides a larger space of feasible passive mechanics that cannot be explored by typical passive elements. Furthermore, the design is low profile and packaged in a small, anthropomorphic form factor. We present an example application of this decoupling mechanism that stores energy from heel-strike and returns this energy in the terminal stance phase of gait. Our approach to recycle collision work may, in part, address some of the deficits associated with use of typical passive prostheses and have beneficial effects on gait biomechanics. In this paper, we describe the design and characterization of the Decoupled Energy Storage and Return (DESR) ankle prosthesis mechanism and validate the efficacy of the new design by benchtop evaluation. In addition, we introduce other possibilities enabled by this decoupling mechanism, which may be pursued as future work.

## Concept

**Figure 1.**
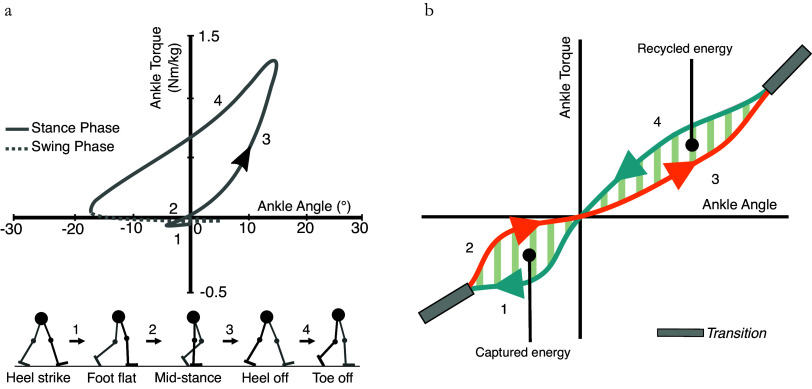
(a) Key positions in the stance phase and average torque–angle curve for able-bodied subjects during level ground walking at a natural speed, from Bovi et al. (2011). (b) A passive approximation of the healthy torque–angle behavior, defined by two distinct nonlinear torque–angle curves. Energy is captured early in the stance phase and recycled in order to enhance the push-off during late stance. A positive angle represents dorsiflexion and a negative angle represents plantarflexion.

To address some limitations of conventional passive prostheses and improve passive emulation of healthy human ankle mechanics (Figure 1a) we propose to reproduce stance phase mechanics using two distinct nonlinear torque–angle curves. To be passively feasible, the net work done by the mechanism must be zero. Reproducing the stance phase with multiple torque–angle curves enables a wider range of possible ankle mechanics in passive prostheses (e.g., the zero-torque equilibrium angle of the ankle joint can be dorsiflexed during the swing phase of gait, independent of the stance phase equilibrium angle, to achieve greater toe clearance). In this paper we focus on a specific application of the proposed concept to emulate biological ankle function which typically produces net-positive work at push-off. Reproducing gait with two different torque–angle curves allows energy from heel-strike collision to be stored, and selectively returned in terminal stance phase to enhance push-off (Figure 1b), an idea previously proposed by Collins and Kuo (2010).

## DESR Design

### VSPA Cam-based Transmission

**Figure 2.**
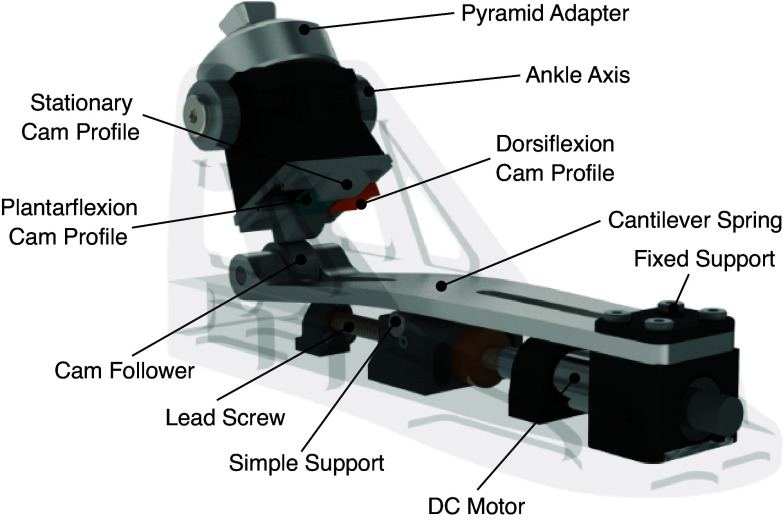
The variable-stiffness prosthetic ankle–foot (VSPA) with Decoupled Energy Storage and Return cam-based transmission. A rotation of the ankle joint causes deflection of a propped cantilever spring via a cam-based transmission. The cam profiles can be shaped to achieve custom torque–angle curves. As in the single cam-based transmission VSPA foot, the stiffness of the foot could be modified by using a small motor to move a simple support under the spring. This functionality was left in the presented prototype for adjustability, but stiffness changes were not explicitly tested in this work.

The VSPA foot (Shepherd and Rouse, 2017b) proposed a cam-based transmission to achieve a prosthesis design capable of nonlinear and customizable torque–angle curves. The cam profile and cam follower (i.e., cam-based transmission) mechanically link the ankle axis of rotation to the cantilever spring (Figure 2). Rotation of the ankle joint deflects the cantilever spring, allowing energy to be stored and released. The shape of the cam profile governs the amount of elastic energy stored in the spring for a given ankle joint rotation. Higher energy storage in the spring results in a larger normal force between the cam profile and cam follower. Importantly, this normal force is only affected by the magnitude of rotation and not the vertical forces placed on the prosthesis. By modifying the shape of the cam, a range of nonlinear torque–angle curves can be generated to achieve the desired ankle mechanics.

### VSPA Variable Stiffness

The torque–angle curve of the VSPA foot (Shepherd and Rouse, 2017b) can be adjusted by re-position the simple support (Figure 2), causing general modulation of the overall stiffness. A small motor and lead screw in the keel can re-position the simple support to adjust the translational stiffness of the cantilever spring. This modulation can be performed during the swing phase of gait, in order to not overload the motor and prevent energy injection into the gait cycle. This stiffness modulation provides versatility to take human preference (Shepherd et al., 2018) and various ambulatory tasks into consideration (Bovi et al., 2011).

### DESR Mechanism Description

The proposed DESR mechanism utilizes a similar cam-based transmission as the VSPA foot (Shepherd and Rouse, 2017b). In this work, we largely assume a fixed slider position, approximately halfway along its range of motion; however, this mechanism is capable of modifying the overall stiffness step-to-step, similar to the VSPA foot design.

**Figure 3.**
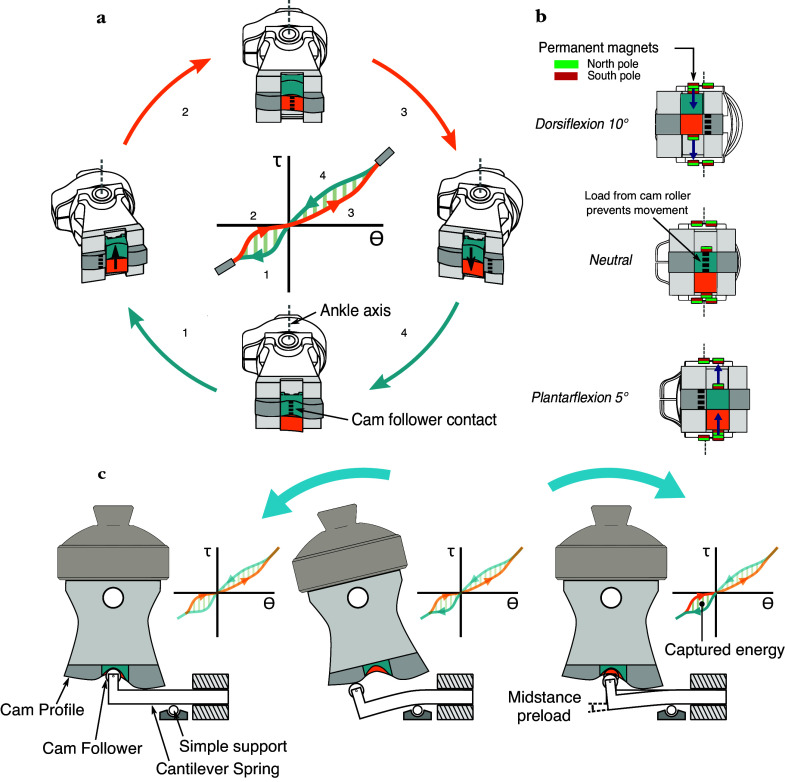
(a) The two cam profiles (plantarflexion cam colored in blue and dorsiflexion in orange) switch based on the ankle angle during stance. The cams are able to freely slide in the groove when the cam follower contacts the stationary cam profile (colored in gray). (b) Magnets attached to the cams and the frame of the prosthesis force the cams to switch when the ankle angle, and thus the cam follower, enter transition regions (>5° plantarflexion or >10° dorsiflexion). (c) Following heel strike, as the ankle moves from plantarflexion through dorsiflexion and the cams switch, the dorsiflexion cam is engaged with the cantilever spring preloaded. This preload enables collision energy to be captured at midstance and returned during late stance in order to enhance the push-off.

To model the stance phase of gait using two different torque–angle curves, the DESR mechanism uses two separate cam profiles which automatically switch at specified ankle angles (Figure 3a). The switchable cam profiles are mechanically linked and can slide freely in a groove when the cam follower is contacting the stationary cam profile (i.e., when the switchable cams are unloaded). We will term the cam that is active as the ankle is moving toward peak dorsiflexion during walking (Phases 2 and 3) the *dorsiflexion cam* and the cam that is active as the ankle is moving toward peak plantarflexion (Phases 1 and 4) the *plantarflexion cam.*

Small permanent neodymium magnets placed on the frame and the sides of the switchable cams are responsible for jogging the cam assembly mediolaterally, swapping which cam is active (i.e., responsible for ankle torque–angle mechanics). This switch occurs when the cam follower enters a transition region, and the switchable cams become unloaded from the cam follower (Figure 3b). The transition regions were defined at >5° plantarflexion and >10° dorsiflexion; we have previously found these values to be within the expected range of motion when subjects walk with the VSPA foot at their preferred stiffness (Shepherd et al., 2018). The values reported by Shepherd et al. (2018) and the able-bodied ankle kinematics at various walking speeds (Bovi et al., 2011) lead us to believe that this mechanism is suitable for the majority of users.

The mathematics governing the relationship between a single cam profile and torque–angle mechanics were previously described in Shepherd and Rouse (2017b) and extended to account for the second cam profile. A second cam profile can be added to the transmission system under the constraint that the total energy stored and released by cam profiles are equivalent. The full mathematical derivation is presented in the Appendix. The main extension to the mathematics was to account for the additional preload of the spring at midstance, which is a function of the spring stiffness and stored energy during early stance (i.e., collision work). (Figure 3c).

Although a wide range of nonlinear torque–angle curves can be generated, there are some constraints on the space of feasible mechanics caused by the nonzero cam follower radius. The cam shape is generated from the desired mechanics by first calculating a progenitor curve (i.e., the cam shape is first generated by modeling the cam follower as a single point), and then offsetting this curve by the radius of the cam follower (Figure 3c). To prevent singularities/discontinuities in the final cam profile, the absolute value of the offset distance should never exceed the minimum radius of curvature in concave regions of the progenitor curve (Wallner et al., 2001). This requirement limits the amount of energy that can be recycled, although this can be improved by increasing the spring stiffness, increasing the distance between the cam follower and ankle axis, or decreasing the radius of the cam follower.

To enable maximum control over the cam profiles to store maximum energy, the torque–angle curves were described by non-uniform rational basis splines (NURBS). NURBS allow defining endpoint tangents, which were required to be the same for both torque–angle curves, and modifying knot weights to adjust the curves in a more controlled manner. In particular, this allowed tight control over the minimum radius of curvature, thus preventing self-intersecting cam profiles. The torque–angle curves were iteratively modified to have equal amounts of captured and recycled energy (see Appendix).

The cams were constructed via wire electrical discharge machining (wire EDM). Tolerances were defined to minimize gaps between the switchable and stationary cams, while keeping enough clearance for low-force switching. The cams were hardened to 60 Rockwell C to prevent plastic deformation due to high compressive forces in the interface with the cam follower. This hardening was done prior to the wire EDM cutting to achieve a higher machining accuracy and prevent shape distortion upon hardening.

## Experimental Methods

The torque–angle curves of the physical prototype were experimentally determined using the neurobionics rotary dynamometer (motor: BSM90N3150AF, Baldor, Fort Smith, AR, and six-axis load cell: 45E15A M63J, JR3, Inc., Woodland, CA). Torque and angle data were sampled at 1,000 Hz. The pyramid adapter of the prosthesis was rigidly attached to the dynamometer and the bottom of the foot was clamped to a rotating foot plate.

The support slider position under the spring could be adjusted in the fore-aft direction with a DC motor, allowing adjustment to the overall stiffness. The range of motion of the slider was 55 mm, with 0 defined as the most anterior (least stiff) position. The primary slider position, the position around which the cam profile is designed, was chosen at 33 mm to enable a large range of stiffness variation. The experiments were performed for a range of slider positions, but only the results for the primary slider position are presented here. To ensure the transition of cam profiles, the prosthesis was tested for the range of 10° plantarflexion and 12° dorsiflexion.

To determine the speed of switching between cam profiles, the ankle was clamped to a testbed, and the pylon manually adjusted to the appropriate transition angles to initiate the cam transitions. A total of 50 transitions (25 each direction) were recorded using a high frame rate video (240 FPS). Transition time was measured by counting frames where there was a difference in the transverse position of the cam between previous and/or subsequent frames. A video illustrating the automatic switching between cam profiles is provided in the Supplementary Material.

## Results

**Figure 4.**
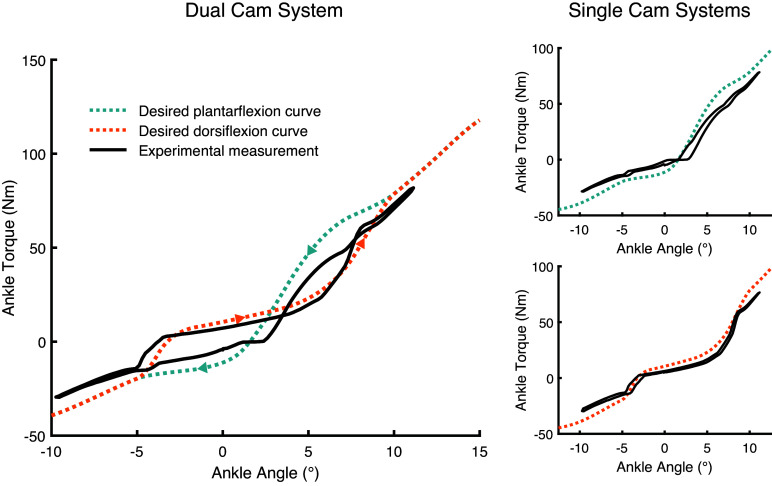
Comparison between theoretical and experimentally measured torque–angle curves for the DESR dual cam system and each cam individually. A positive angle is dorsiflexion and a negative angle is plantarflexion.

Despite the increase in push-off due to recycling energy stored during early stance phase, the experimentally determined push-off work was lower than the expected theoretical work for the DESR mechanism (Table 1). Our model predicted a 15.2% increase in energy returned to the ankle during push-off (i.e., the energy released during Phase 4 should be 15.2% larger than the energy stored during Phase 3 Figure 1b), but experimentally, we observed a net loss of 2.1% meaning that less energy was released during push-off than was stored during mid- to late stance (Figure 4 and Table 1). Testing the plantarflexion or dorsiflexion cam profiles independently yielded energy losses of over 10% for each cam profile (Figure 4). While there was not a net positive increase in energy release for the DESR mechanism, the extra energy stored and released did offset much of the hysteresis losses, leading to considerably lower hysteresis losses than the average 25% seen in commercially available ESR feet (Geil, 2001; Table 1). The magnetic mechanism successfully caused transitions between cams to occur in all trials. Transitions to the plantarflexion cam took 39 ± 32 ms (mean ± standard deviation [SD]) and transitions to the dorsiflexion cam took 61 ± 40 ms.

**Table 1.** Energy stored during dorsiflexion and returned at push-off for the Decoupled Energy Storage and Return (DESR) mechanism, and commercially available ESR feet Geil (2001).

	DESR		ESR	
	Theoretical	Measured	Theoretical	Measured
Energy stored over dorsiflexion (J)	8.1	7.0	n/a	2.1–8.1
Energy returned during late stance (J)	9.1	6.9	n/a	1.6–5.8
Energy returned (%)	115.2	97.9	≤100	68–82

## Discussion

In this paper, we introduced and validated the mechanics of a new, passive mechanism which is capable of decoupling the elastic properties of an ankle–foot prosthesis. A dual cam-based transmission system was leveraged to elicit independent, nonlinear torque–angle responses in the prosthesis, depending on the phase of gait. To validate the mechanism, we designed a prototype to recycle collision energy stored during early stance heel-strike to enhance terminal stance push-off work. In this prototype, we also assessed a custom designed magnetic switching system capable of passively interchanging cam profiles at specific points in the gait cycle.

**Figure 5.**
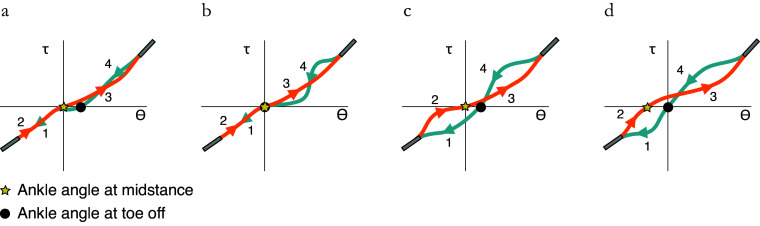
Outcomes of different DESR mechanisms: (a) dorsiflexed neutral ankle angle (b) varied release rate of energy in late stance (c) increased foot clearance and energy storage (d) increased energy storage. A positive angle is dorsiflexion and a negative angle is plantarflexion.

The dual cam design enables the fusion of beneficial aspects of different cam profiles to create highly customizable energy storage and return profiles. The prototype presented in this paper illustrated two potential benefits: providing additional energy during push-off to decrease hysteresis losses by 8%, and using cams with different equilibrium angles such that the ankle is dorsiflexed by 1.6° in swing. In comparison, a healthy ankle joint dorsiflexes by 5.1° during the swing phase (Bovi et al., 2011), and conventional ESR feet dorsiflex by 0°. Differently designed DESR systems could alter the energy storage and ankle angle properties in a variety of ways. These effects include changing the equilibrium ankle angle (Figure 5a), adapting the release rate of the stored energy during push-off (Figure 5b), dorsiflexing the ankle and simultaneously increasing the range of motion along which energy can be stored (Figure 5c), or creating a slightly plantarflexed neutral ankle angle to increase captured collision energy (Figure 5d). Similar cam-based DESR mechanisms could be used in other applications that employ nonlinear springs or clutches. For example, a passive ankle exoskeleton employing a clutched spring improved the energetic cost of walking by 7% (Collins et al., 2015). Similarly, a passive hip exoskeleton that improves the energetic cost of running with a linear spring could more accurately follow the relationship between hip moment and difference in hip angles with decoupled, nonlinear springs (Nasiri et al., 2018).

Our specific implementation of the decoupling mechanism was designed to capture energy normally dissipated at heel-strike and loading response and return it during late stance. Theoretically, this application of the dual-cam system should have provided net positive push-off power, but this was not the case. The DESR mechanism returned additional energy during push-off, but the amount was not enough to overcome hysteresis losses of the mechanical system. These unexpected losses were likely due to imperfect elasticity in the ankle’s spring and frame, and during cam deformations, particularly during transitions between cams where very small gaps or step changes in height could cause stress concentrations or energy loss. Energy may also have been lost due to flexing of the testing apparatus. Another potential source of error can be caused by slippage between the testing apparatus and the DESR mechanism. Other researchers have used Teflon sheets to decrease this friction in order to minimize hysteresis losses (Geil, 2001).

While the prototype did not meet the theoretical energy return expectation, the DESR mechanism exhibited much more efficient energy return than a single cam profile or comparable ESR prostheses. In a study of 11 commercially available prosthetic feet Geil (2001) found that hysteresis losses in the prostheses ranged from approximately 18% (Dynamic Plus by Ottobock, Duderstadt, Germany) to 32% (TruStep by College Park, Warren, MI). Ten of these prosthetic feet were tested with a foam cover, which likely contributed to the hysteresis losses (Van Jaarsveld et al., 1990). Both the TruStep foot and the DESR mechanism stored approximately 7 J during stance phase, but the TruStep foot lost approximately 2 J (Geil, 2001) per step, compared to the DESR’s loss of only 0.1 J per step. The DESR mechanism’s high energy return efficiency is promising, and with design modifications, could potentially achieve energy return closer to the theoretical maximum of 115.2% efficiency for push-off work. These results demonstrate not only the ability of the mechanism to decouple the elastic properties of the prosthesis, but also the potential of our DESR design to capture and return more energy during push-off than is observed in conventional ESR feet.

To evaluate the efficacy of the magnetic switching system, we recorded the time required for each cam to slide into place once the cam roller contacted the transition surface of the ankle. The transition from dorsiflexion cam to plantarflexion cam took longer than vice versa (61 ± 40 ms compared to 39 ± 32 ms), and both transition times had high variability, likely due to inconsistencies in angular velocity at transition during the manual testing. The switching time is critical for successful performance; if the cam is unable to switch during the time the cam roller is in contact with the transition surface, then the active cam roller may or may not change (it will ride along the highest profile), and stresses will be substantially increased. Further study will determine whether the cam profiles switch fast enough to accommodate a variety of cadences and speeds, and may depend on the specific range of motion of the user. Understanding how the DESR mechanism performs during clinical testing is an important area for future work.

It is also possible to design the switching mechanism such that it does not automatically switch between cam profiles, but could instead be triggered by the user. This could allow the use of multiple cam profiles, where each cam is only engaged during the desired task (e.g., one cam profile could be designed with a highly dorsiflexed neutral angle to increase toe clearance during stair or ramp ascent). Quasi-passive designs employing this technique could benefit from greater customization, without adding the weight, cost, or audible noise of fully powered systems. More research is needed to explore the applications of both an automatic and user-triggered switching system in conjunction with the multiple implementations of the decoupling mechanism in order to create ankle–foot prostheses capable of adapting to different environments and use-cases.

Limitations of this study include testing the DESR mechanism with below-knee amputees. The benchtop characterization in this work is performed to determine the mechanics of the device and verify the automatic switching, under loads similar to amputee gait. This was done in a controlled environment with a fixed range of motion and angular velocity. Further work is required to investigate how amputees experience the DESR mechanism during gait.

## Conclusion

This paper proposed a new mechanism to decouple the elastic mechanical properties in passive ankle–foot prostheses. Two nonlinear torque–angle curves were used to design a dual cam-follower transmission, allowing for a unique customizability of the ankle mechanics. We presented one implementation of this mechanism which was designed to separate the paths of energy storage and return. While this application of the mechanism was able to successfully decouple energy storage and return properties, it was not successful in providing net-positive mechanical work during the push-off. Newer generations of this application should focus on minimizing the hysteresis effects seen in the results in order to increase the amount of energy returned during late stance. Future work will also focus on tailoring applications of the decoupling mechanism toward specific patient preferences and performing multiple ambulatory tasks.

## Figures and Tables

**Figure A1.**
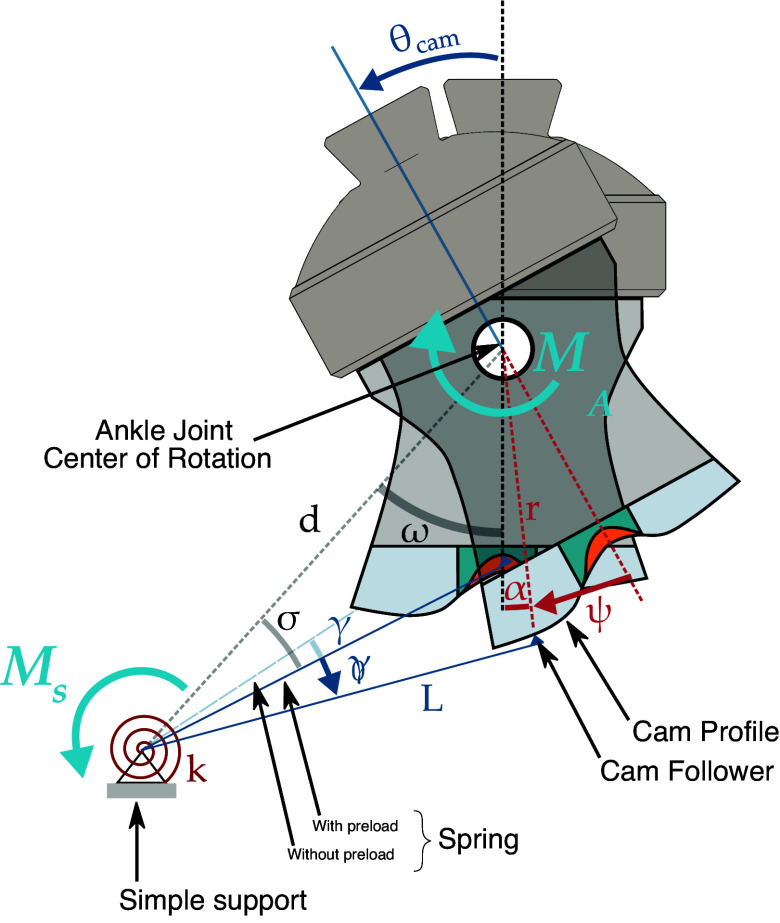
Energy stored in the beam spring “L” (modeled as a rotational spring with a fixed simple support underneath the spring) can be used to perform work at the ankle joint. The ankle joint and spring are mechanically linked via a cam profile and a cam follower, which is modeled as a point. When the spring is bent a torque (𝑀_S_) is created around the simple support, which results in a response torque (*𝑀_𝐴_*) at the ankle joint. The energy stored at the ankle joint is equal to the energy stored in the frame and the spring combined. Thus, the rotation of the ankle joint is a combination of the frame compliance (𝛿) and the rotation of the cam profile (𝜃_𝑐𝑎𝑚_). With these variables and the fixed geometry (shown in gray) the polar coordinates (*r*,*𝜓*) can be calculated to determine the cam profile shape. Finally, the cam radius must be used to determine an offset curve, which defines the shape of the actual cam profile.

## Data Availability

Data and processing code are freely available at https://doi.org/10.5281/zenodo.4813707.

## Supplementary material

For supplementary material accompanying this paper visit https://doi.org/10.1017/wtc.2021.7. click here to view supplementary material

## Appendix

### Cam Design

#### Nomenclature

This appendix describes the mathematical relationships used to determine the shape of a cam profile in order to achieve the desired ankle mechanics (i.e., torque–angle curve). The symbols and variables used throughout the Appendix to describe the mathematics are defined in Table A1.

**Table A1.** List of symbols used in the Appendix.

M*_𝑆_*	Torque at the interface of the spring and simple support
M*_𝐴_*	Torque at the ankle joint
d	Absolute distance between the ankle joint and the interface of the spring and simple support
L	Absolute distance between the cam follower and the interface of the spring and simple support
r	Cam radius
*𝜃*𝑐𝑎𝑚	Rotation of the cam profile
*σ*	The angle between d and L at the neutral ankle angle *𝜃_𝑐𝑎𝑚_*
*𝛾*_0_	Angular deflection of the rotary spring, due to a preload
*𝛾*	Rotation of the rotary spring
k	The rotational stiffness of the cantilever beam spring, around the simple support
*ω*	Angle between d and the vertical axis
*𝛼*	Angle between r and the vertical axis, caused by the horizontal displacement of the cam follower due the rotary motion of the spring
*𝜓*	Effective cam profile angular deflection, accounting for *𝛼*
*𝛿*	Change in ankle angle due to elastic deflection of the frame

##### Virtual work

The principles of virtual work are used to obtain the cam profile shape for a given torque–angle curve, considering it is a conservative energy system with no losses (Figure A1). The cam follower is modeled as a point, but the final cam profile curve will be offset by using the cam follower radius and applying the theorem of parallel curves. The rotation of the ankle joint (*𝜃*_𝑐𝑎𝑚_) results in a force between the cam profile and the cam follower. This generates a moment (*𝑀_𝑆_*) around the simple support due to the deflection of the spring, which is modeled as a rotary spring. For a rigid prosthesis body frame with infinite stiffness, it can be assumed that the potential energy stored into the spring, due to an angular deflection (*𝛾*) and moment (*𝑀_𝑆_*), should be equal to the work done at the ankle joint. However, experiments indicated some form of elastic deformation of the frame due to the response torque (*𝑀_𝐴_*) around the ankle joint. This frame compliance (*𝛿*), or elastic deformation of the frame, should be accounted for in the calculations. The mathematical model assumes a deflection of the frame proportional to the torque at the ankle joint. Previously this stiffness was experimentally determined to be 1,200 Nm/rad for the original VSPA foot (Shepherd and Rouse, 2017b) and 2,000 Nm/rad for the redesigned frame.

##### First Cam Profile Equations

This section provides the equations and assumptions used to mathematically determine the shape of a single cam profile, for a given torque–angle curve. A detailed description on the single cam-based transmission design is provided in Shepherd and Rouse (2017b).

The torque–angle curves mentioned in the paper refer to the torque (*𝑀_𝐴_*) and the angular deflection (*𝜃*_𝑐𝑎𝑚_) of the ankle joint. The ability to perform work around the ankle joint depends on the amount of energy stored in the prosthesis body frame and the cantilever beam spring combined.

The energy stored at the ankle joint minus the energy stored in the frame equals the energy stored in the spring (Equation 1):

(1)

At the neutral ankle position of *𝜃* = 0 there is no torque (*𝑀_𝐴_*) around the ankle joint. At this position, the spring is preloaded and bent by an angle of *𝛾*_0_ to remove play between the components. The energy equation of the spring can be described using the rotational stiffness of the spring (*k*) and its angular deflection (*𝛾*).

The equilibrium position is *𝜃* = 0. Dorsiflexion and plantarflexion are solved separately with 0 as the lower limit of integration (Equation 2):

(2)

The spring equation can be further integrated to solve for (*𝛾*).

Integration of the left side of the equation, using boundary conditions *𝛾* = 0 at *𝜃* = 0 (Equations 3 and 4):

(3)

Solving for Equation (3) results in a constant of integration (c) of zero. Equation (3) can now be rewritten to solve for *𝛾* as a function of *𝜃*:

(4)

The ability to describe *𝛾* as a function of *𝜃* allows us to describe the cam radius (*r*) as a function of *𝜃* using the law of cosines.

Polar representations of the cam profile (Equations 5 and 6):

(5)

With a known *r*, the shape of the cam profile can be described using the polar representations (*r*, *ψ*).

(6)

The spring is modeled as a rotary spring, causing the cam follower to move in both the horizontal and vertical direction upon rotation of the ankle. The angle *𝛼* caused by the horizontal displacement of the cam follower can be derived using the law of sines.

Angular deflection from vertical (Equation 7):

(7)

##### Second cam profile equations

This section provides the equations for modeling a second cam profile for the dual cam-transmission. It uses the same mathematical steps as Equations (2)–(7), but accounts for adaptations required due to the changed ankle mechanics. The key difference is that the second cam profile enables energy storage in the spring at midstance, implying that the spring is bent at *𝜃* = 0. The number “2” after the symbols in the following equations indicates values for the second cam profile (e.g., *𝛾*2 and *𝛿*2).

The energy in the spring (Equation 8):

Here, *𝛾*_20_ is an added preload at midstance that depends on the amount of energy stored in the spring at midstance.

(8)

Preload equation for captured energy (Equation 9):

Here, ES is the energy stored in the spring at midstance.

(9)

Value of *𝛾*_20_ (Equations 10 and 11):

(10)

If *𝛾*_20_ = *𝛾*_0_ is assumed as a boundary condition, the integration constants are found to be 0, and we can solve for *𝛾*_20_ (Equation 11):

(11)

Spring deflection (Equations 12 and 13):

With the calculated additional preload (*𝛾*_20_), Equation (8) can be further integrated to determine the spring deflection caused by the second cam profile (Equation 12):

(12)

By taking *𝜃* = 0 as a boundary condition, the integration constant is found to be 0 again (Equation 13):

(13)

Polar representation of the second cam profile (Equations 14–16):

(14)

(15)

(16)

Angular deflection from vertical (Equation 17):

(17)
