# Supplementary material for: A passive mechanism for decoupling energy storage and return in ankle–foot prostheses: A case study in recycling collision energy
Source: Wearable Technol. 2021 Jul 28;2:e9. doi: 10.1017/wtc.2021.7 (PMC10936356; doi:10.1017/wtc.2021.7)
Supplement: Supplementary file 1 [file wtcsup.zip › S2631717621000074sup002.docx]

**Video Legend**

Decoupled Energy Storage and Return (DESR) prosthesis prototype shown. As the ankle joint rotates, a magnet-based switching system alternates the engagement of the two cam profiles, thereby enabling different torque-angle and energy storage mechanics associated with each cam profile.
